# Comparison of the Analgesic Efficacy of Ultrasound-Guided Transperineal Approach Using Pudendal Nerve Block Versus Caudal Block in Children Undergoing Urological Surgeries: A Randomized Controlled Trial

**DOI:** 10.7759/cureus.74244

**Published:** 2024-11-22

**Authors:** Varun K Singh, Anju Gupta, Amita Gupta, Pratiti Choudhuri, Maansi Gangwal

**Affiliations:** 1 Department of Anesthesiology, Critical Care and Pain Medicine, Vardhman Mahavir Medical College and Safdarjung Hospital, New Delhi, IND; 2 Department of Anesthesiology, Critical Care and Pain Medicine, All India Institute of Medical Sciences, New Delhi, IND; 3 Department of Pediatrics and Neonatology, Vardhman Mahavir Medical College and Safdarjung Hospital, New Delhi, IND

**Keywords:** caudal block, pediatrics, perineal surgeries, postoperative analgesia, pudendal nerve block, regional anesthesia, ultrasound guided regional anesthesia

## Abstract

Introduction: Caudal block is an effective regional anesthesia technique for perineal surgeries but is associated with various adverse effects. Recently, pudendal nerve block has emerged as a promising alternative for these procedures. This study assessed the effectiveness of a novel transperineal technique for ultrasound-guided pudendal nerve block and compares it with ultrasound-guided caudal block for perineal surgeries in pediatric patients.

Methods: The study included 60 American Society of Anesthesiologists (ASA) Physical Status Classification System grade I/II children aged 1-12 years and scheduled for elective perineal operations under general anesthesia. Patients were randomly allocated to two equal groups: Group C (ultrasound-guided caudal block) and Group P (ultrasound-guided pudendal nerve block). The groups were evaluated for time to first rescue analgesia (primary outcome), block performance time, total analgesic consumption, Face, Legs, Activity, Cry, and Consolability (FLACC) scores, time to achieve a Post-Anesthetic Discharge Scoring System (PADSS) score of 9, and any complications.

Results: The median interquartile range (IQR) first rescue analgesia time was significantly higher in the pudendal block (17.5 (17-18) hours vs 4.65 (4.5-4.77) hours; p<0.001). The median (IQR) dose of postoperative analgesia (diclofenac) required was similar in the pudendal block group and caudal group (10 (0-10) mg vs 10 (0-20) mg; p=0.290). The median (IQR) FLACC scores at "zero" hour post-surgery were 2 (2-2) and 2 (1-2) in the pudendal and caudal groups, respectively (p=0.052). The median (IQR) PADDS score was significantly higher in the pudendal group (10 (9.25-10) vs 9 (8-9); p<0.001). The time to reach a PADSS score of 9 was significantly longer in the pudendal group (21.4±3 vs 14.9±4.8 hours; p<0.0001). There were no complications in either group.

Conclusions: The findings of this study suggest that pudendal nerve block provides longer-lasting analgesia, lower pain scores, and faster readiness for discharge though with a similar analgesic consumption compared to caudal block. These results indicate the potential of ultrasound-guided pudendal nerve blocks as a beneficial and safe alternative to caudal block for perineal procedures in children.

## Introduction

Regional anesthetic techniques are often used in order to improve postoperative pain management, reduce the need for parenteral opioids, and enable effective pain control during pediatric procedures. In children having urological surgeries, caudal epidural block is the most commonly used technique as part of multimodal analgesia [1]. However, caudal block, due to its broader anesthesia, has some notable limitations like urinary retention and leg weakness [2]. Furthermore, the association of caudal blocks with increased rates of urethrocutaneous fistula following hypospadias surgery is a matter of concern [3]. Hence, nerve stimulation-guided transperineal pudendal nerve block is increasingly used as an alternative technique to caudal analgesia during pediatric perineal surgery [4-6]. However, this blindfolded approach has an inherent danger of intravascular injection or rectal puncture. More recently, research has shown that ultrasound-guided caudal block may be a valuable strategy for increasing the success rate of blocks, providing prolonged postoperative analgesia, effective and denser anesthesia with lesser dose, and fewer adverse effects [7-9].

In clinical practice, the effectiveness of pudendal nerve block is widely established. Although several research studies reference this approach in adult anesthesia, there are few in pediatric anesthesia. In children, it has recently been proposed as an efficient alternative to caudal analgesia for perineal procedures, e.g., hypospadias repair, circumcision surgery, etc. [10]. Few studies in the past have explored the utility and safety of the ultrasound-guided pudendal block for surgeries in children, and it is yet to be established as a routine technique. The feasibility and effectiveness of ultrasound-guided pudendal block in the pediatric population were evaluated in a previous study by Gaudet-Ferrand et al. and Cadavid et al. [7,10]. Ahmed et al. reported significantly better analgesia and analgesics requirement levels in the children who underwent hypospadias surgery when ultrasound-guided pudendal nerve block was applied [11]. In contrast, Okoro et al. reported a 7.5% rescue morphine rate among the ultrasound-guided pudendal block group, which was higher than the conventional neuro-stimulation-guided technique of the pudendal block group [12]. However, no studies have compared the efficacy of ultrasound-guided pudendal block with caudal block in Indian settings.

The present study was designed to compare the duration of analgesia (time for a request for the first rescue analgesic drug from the time of giving the block) provided by pudendal nerve block with that of the caudal block using ultrasound guidance in the pediatric population undergoing surgeries under general anesthesia. Other outcomes included block performance time and puncture success, analgesic consumption, pain scores, recovery time, and complications.

## Materials and methods

Study design and setting

This prospective interventional randomized comparative trial was conducted at the Department of Anesthesia and Intensive Care, Vardhman Mahavir Medical College and Safdarjung Hospital, New Delhi, India, following the approval from the institute's Institutional Ethics Committee (approval number: IEC/VMMC/SJH/Thesis/2019-10/236) and was registered with the Clinical Trials Registry - India (CTRI) (REF/2019/12/030268).

Study participants

The study enrolled children in the age group ranging from one to 12 years with an American Society of Anesthesiologists (ASA) Physical Status Classification System grade I/II who were scheduled for elective urethral surgeries.

Sample size

The study by Ravi et al. observed that the average time to first rescue analgesia was 219.6±48.4 min in the ultrasound-guided caudal group in children aged 3-12 years [13]. Taking these values as a reference and assuming a 15% higher time to first rescue analgesia in pudendal nerve block, the minimum required sample size was 27 (power of study=80% and level of significance=5%). To further reduce the margin of error, the final sample size was taken as 60 participants, with 30 participants in each group.

Intraoperative period

**Figure 1:**
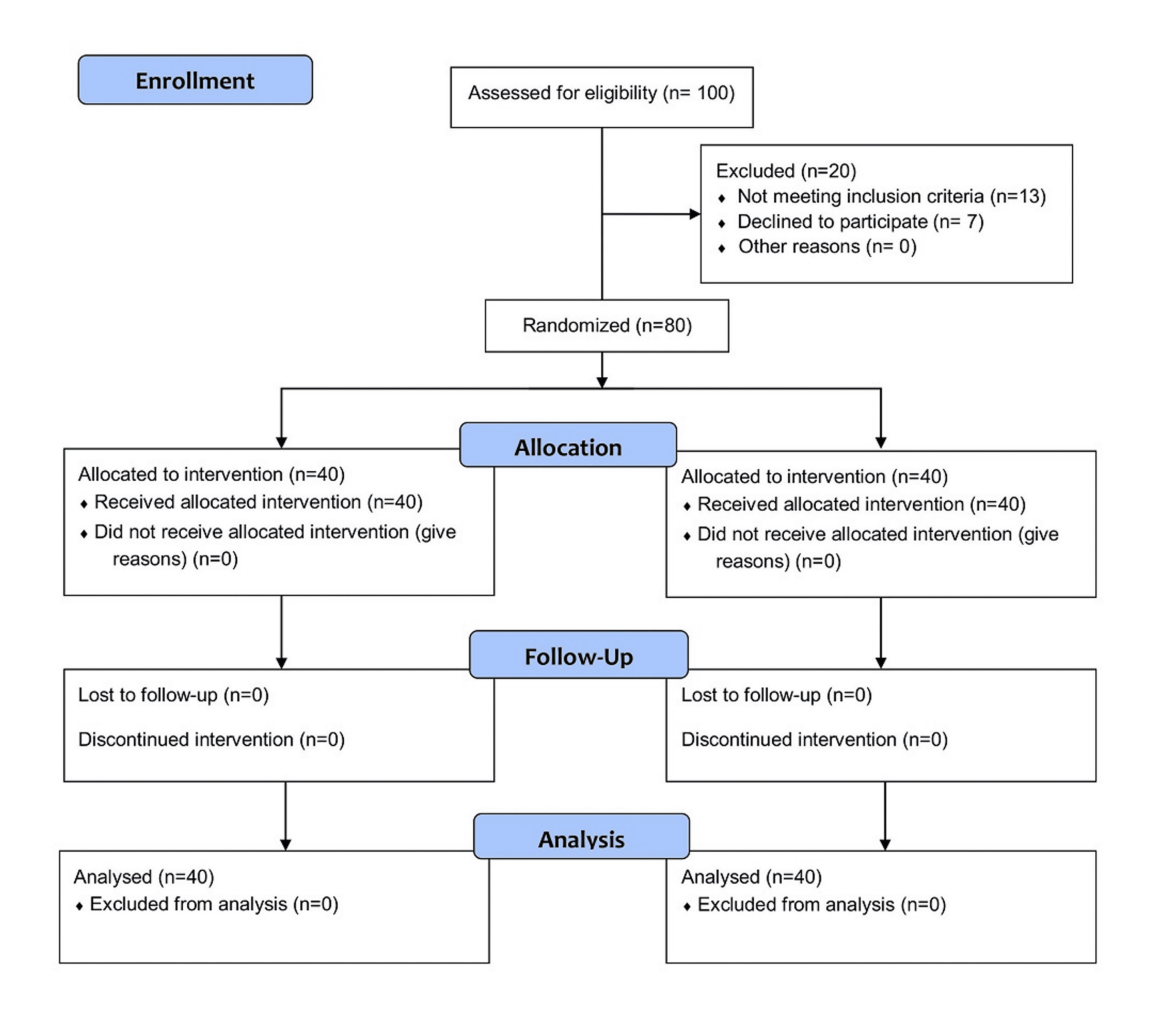
CONSORT flow diagram detailing the enrollment, allocation, and follow-up of patients CONSORT: Consolidated Standards of Reporting Trials

Parental informed consent and participant assent (from children over seven years) were obtained before the study commenced. Preanesthetic check-up was done, and children with any coagulation disorder, rash or infection at the injection site, and allergies to local anesthetics and patients with apparent anatomical anomalies (sacral dimple, tethered cord, and other sacral malformations) or any neurological or spinal disorders were excluded. Patients were randomized by the use of computer-generated random numbers (in blocks of 10 patients per group) using the opaque sealed envelope technique into one of two treatment groups: Group C (n=30) (ultrasound-guided caudal block) and Group P (n=30) (ultrasound-guided pudendal nerve block). The patients, the parents, and the person assessing the postoperative outcomes needed to be made aware of which group they belonged to (Figure 1).

The primary outcome assessed was the duration of analgesia. Other outcomes included procedural time, intraoperative hemodynamic parameters, overall block success rate and postoperative pain scores, analgesic consumption, and complications.

Block procedures

Anesthesia was induced using an injection of propofol 2-3 mg/kg in children with a pre-existing intravenous cannula or a facemask with 7-8% sevoflurane, 50% air, and 50% oxygen until the patient became unconscious. Vascular access was obtained following loss of consciousness in children induced with sevoflurane, and muscle relaxation was achieved using vecuronium (0.1 mg/kg). The appropriate size of the ProSeal laryngeal mask airway (LMA) (Teleflex Incorporated, Wayne, PA, USA) was inserted three minutes following this. To achieve a minimum alveolar concentration (MAC) of 1 MAC, anesthesia was maintained using sevoflurane (in 50% nitrous oxide and 50% oxygen). Ventilation was controlled to maintain an end-tidal carbon dioxide between 35 and 45 mmHg.

In the caudal group, the patient was positioned laterally to administer a caudal block. The solution for the block consisted of 0.25% bupivacaine at a dosage of 0.75 ml/kg to a maximum volume of 20 ml. Following sterilizing the target area and using a sterile plastic cover and gel on the ultrasound probe, the sacral hiatus was identified adjacent to the sacral cornua using a linear transducer (8-13 MHz) from an M-Turbo ultrasound machine (M-Turbo, SonoSite Inc., Seattle, WA, USA). Adjustments were made to the depth and gain settings to enhance the visual clarity.

The linear array probe was initially positioned transversely at the midline to capture a cross-sectional view of the sacrococcygeal ligament, sacral bone, sacral hiatus, and two cornua. The probe was then rotated 90° to secure longitudinal images of the sacrococcygeal ligament and to position the sacral hiatus between the cornua. A 22-gauge needle used for the caudal block was inserted towards the upper third of the sacrococcygeal ligament. In the pudendal nerve block group, following the sterile preparation of both the probe and the skin, children were placed supine with their hips abducted, their legs flexed, and the soles of their feet touching, resembling a "frog position." Ultrasound images were obtained using a portable ultrasound unit (SonoSite M-Turbo) and an 8-13 MHz 38 cm linear array probe. The ischiorectal fossa is located between the ischium laterally and the rectum medially. The pudendal artery was identified using the color Doppler. After careful skin preparation with alcohol povidone-iodine according to our local hygiene protocol, a 22-gauge 50 mm insulated needle was then introduced at the middle of the superior edge of the probe, in an out-of-plane approach, with an inclination of 15° in the sagittal plane, in an anterior-posterior direction. The position of the needle's tip was identified by direct vision, through the movement of adjacent anatomical structures, or with saline injection.

After a negative aspiration test, 0.25 ml/kg of a solution of 0.25% bupivacaine was injected bilaterally in the ischiorectal fossa near the pudendal artery under real-time ultrasound scanning. The surgery was allowed to proceed 15 minutes after injection.

Block failure was defined as "any rise in heart rate or blood pressure of more than 20% on skin incision. Intraoperatively, fentanyl 1-2 µmcg/kg was supplemented whenever there was a rise in hemodynamic parameters more than 20% above baseline."

Intraoperatively, the duration of surgery, block performance time, success rate, fentanyl consumption, and hemodynamic parameters were recorded. Paracetamol 15 mg/kg was routinely given intravenously to all children eight hourly. At the end of the surgery, anesthesia was reversed, and ProSeal LMA was removed. The Face, Legs, Activity, Cry, and Consolability (FLACC) Behavioral Pain Scale score for 1-7 years of age, Visual Analogue Scale (VAS) score for 7-12-year-old children, and Post-Anesthetic Discharge Scoring System (PADSS) score were recorded postoperatively. Any adverse effects (local pain, hematoma, infection, etc.) were recorded.

Statistical analysis

Categorical variables were presented as counts and percentages, while continuous variables were described using mean±standard deviation (SD) or median. Continuous data was found to be skewed by applying the Kolmogorov-Smirnov test. Hence, the Wilcoxon-Mann-Whitney U test was used to assess the significance of the difference between the two groups. The chi-squared test compared qualitative variables. The Friedman test examined changes in FLACC/VAS over time within each group, and the generalized estimating equations method assessed differences in these changes between the two groups over time. Statistical significance was taken at a p-value below 0.05. Statistical analysis utilized the IBM SPSS Statistics for Windows, V. 21.0 (IBM Corp., Armonk, NY, USA) software.

## Results

The present randomized controlled trial was conducted on 60 pediatric patients undergoing urologic surgery at a tertiary care center in North India. Half of the patients received caudal block, and the other half received pudendal block. Seventy-five patients were assessed for eligibility, of which 10 parents denied consent to participate, three were excluded based on exclusion factors, and surgery was postponed for two due to surgical reasons (Figure 1).

Table 1 enumerates the characteristics of the study participants, revealing no statistically significant differences between the two groups. Hypospadias was the most common diagnosis, and urethroplasty was the most common procedure among the study participants (Table 1).

**Table 1:** Demographic and clinical characteristics of the study participants *Wilcoxon-Mann-Whitney U test; ^†^Fisher's exact test; ^‡^chi-squared test ASA grade: American Society of Anesthesiologists Physical Status Classification System; Group C: caudal block group; Group P: pudendal block group

	Group C	Group P	P-value
Age (years)	6.83±2.85	6.93±3.46	0.847* (U=436.500)
Age			1.000^ ‡ ^(χ^2^=0.000)
2-5 years	11 (36.7%)	11 (36.7%)	
6-12 years	19 (63.3%)	19 (63.3%)	
Sex (male)	30 (100%)	30 (100%)	1.000^‡^(χ^2^=0.000)
Weight (kg)	23.67±6.98	22.60±9.29	0.711* (U=475.500)
Diagnosis			0.637^†^(χ^2^=2.222)
Hypospadias	17 (56.7%)	17 (56.7%)	
Phimosis	8 (26.7%)	10 (33.3%)	
Urethral stricture	3 (10%)	3 (10%)	
Epispadias	2 (6.7%)	0 (0%)	
Surgery			0.925^†^(χ^2^=0.333)
Urethroplasty	19 (63.3%)	17 (56.7%)	
Circumcision	8 (26.7%)	10 (33.3%)	
Urethral calibration	3 (10%)	3 (10%)	
ASA grade			0.237^†^ (χ^2^=3.158)
I	27 (90%)	30 (100%)	
II	3 (10%)	0 (0%)	

The success rate for the first puncture was 93.4% (28/30) in the caudal block group and 73.4% (22/30) in the pudendal block group. The overall block success rate was 90% in the caudal block group and 83.3% in the pudendal block group. The success rate in the first puncture and the overall block were similar between the two groups (Table 2). The caudal block group had significantly shorter block performance time (median=10 minutes) than the pudendal block group (median=16 minutes) and significantly lower intraoperative fentanyl consumption but with similar total postoperative analgesia consumption. Longer first rescue analgesia was found among the pudendal block group than the caudal block group (p<0.05). 

**Table 2:** Association between the study groups and various outcomes *Wilcoxon-Mann-Whitney U test; ^‡^chi-squared test IQR: interquartile range; Group C: caudal block group; Group P: pudendal block group

	Group C	Group P	P-value
Success of the first puncture	N (%)	N (%)	0.083^ ‡ ^(χ^2^=3.000)
Successful	28 (93.4%)	22 (73.4%)	
Not successful	2 (6.6%)	8 (26.6%)	
Block success	N (%)	N (%)	0.704^ ‡ ^(χ^2^=0.144)
Successful	27 (90%)	25 (83.3%)	
Not successful	3 (10%)	5 (16.7%)	
Block performance time (minutes): median (IQR)	10 (9-12)	16 (16-18)	<0.001* (U=0.500)
Total intraoperative fentanyl consumption (µg): median (IQR)	0 (0-10)	0 (0-0)	0.008* (U=593.000)
Time of first rescue analgesia (hours): median (IQR)	4.65 (4.5-4.77)	17.5 (17-18)	<0.001* (U=104.500)
Total postoperative analgesia consumption (mg): median (IQR)	10 (0-20)	10 (0-10)	0.290* (U=519.000)

**Figure 2:**
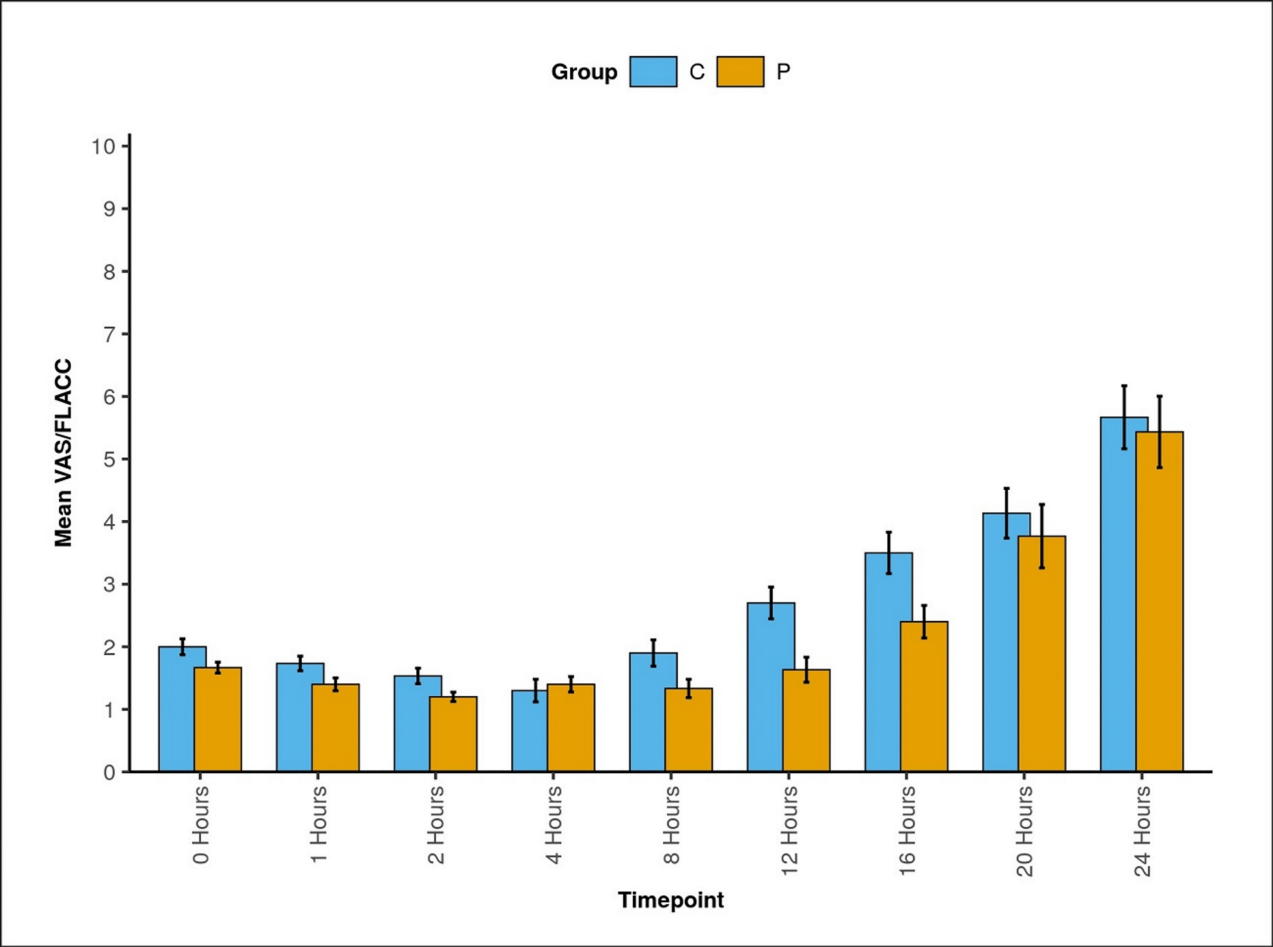
Bar diagram showing the change in FLACC/VAS (along with SD) over time between the two groups Group C: caudal block group; Group P: pudendal block group; FLACC score: Face, Legs, Activity, Cry, and Consolability Behavioral Pain Scale score; VAS score: Visual Analogue Scale score; SD: standard deviation

The two groups differed significantly regarding FLACC/VAS at one, two, eight, 12, and 16 hours (Table 3, Figure 2).

**Table 3:** Comparison of the two groups in terms of change in FLACC/VAS over time SD: standard deviation; Group C: caudal block group; Group P: pudendal block group; FLACC score: Face, Legs, Activity, Cry, and Consolability Behavioral Pain Scale score; VAS score: Visual Analogue Scale score

Time point	FLACC/VAS		
	Group C: mean (SD)	Group P: mean (SD)	P-value (Wilcoxon-Mann-Whitney U test)
0 hours	2.00 (0.69)	1.67 (0.48)	0.052 (U=335.000)
1 hours	1.73 (0.64)	1.40 (0.56)	0.036 (U=577.000)
2 hours	1.53 (0.68)	1.20 (0.41)	0.025 (U=579.000)
4 hours	1.30 (0.99)	1.40 (0.67)	0.476 (U=405.500)
8 hours	1.90 (1.16)	1.33 (0.80)	0.030 (U=590.000)
12 hours	2.70 (1.39)	1.63 (1.10)	0.004 (U=641.500)
16 hours	3.50 (1.81)	2.40 (1.43)	0.010 (U=621.000)
20 hours	4.13 (2.18)	3.77 (2.78)	0.289 (U=521.000)
24 hours	5.67 (2.76)	5.43 (3.13)	0.685 (U=477.500)
P-valuefor change in FLACC/VAS score over time within each group (Friedman test)	<0.001 (χ^2^=124)	<0.001 (χ^2^=124.2)	-

**Figure 3:**
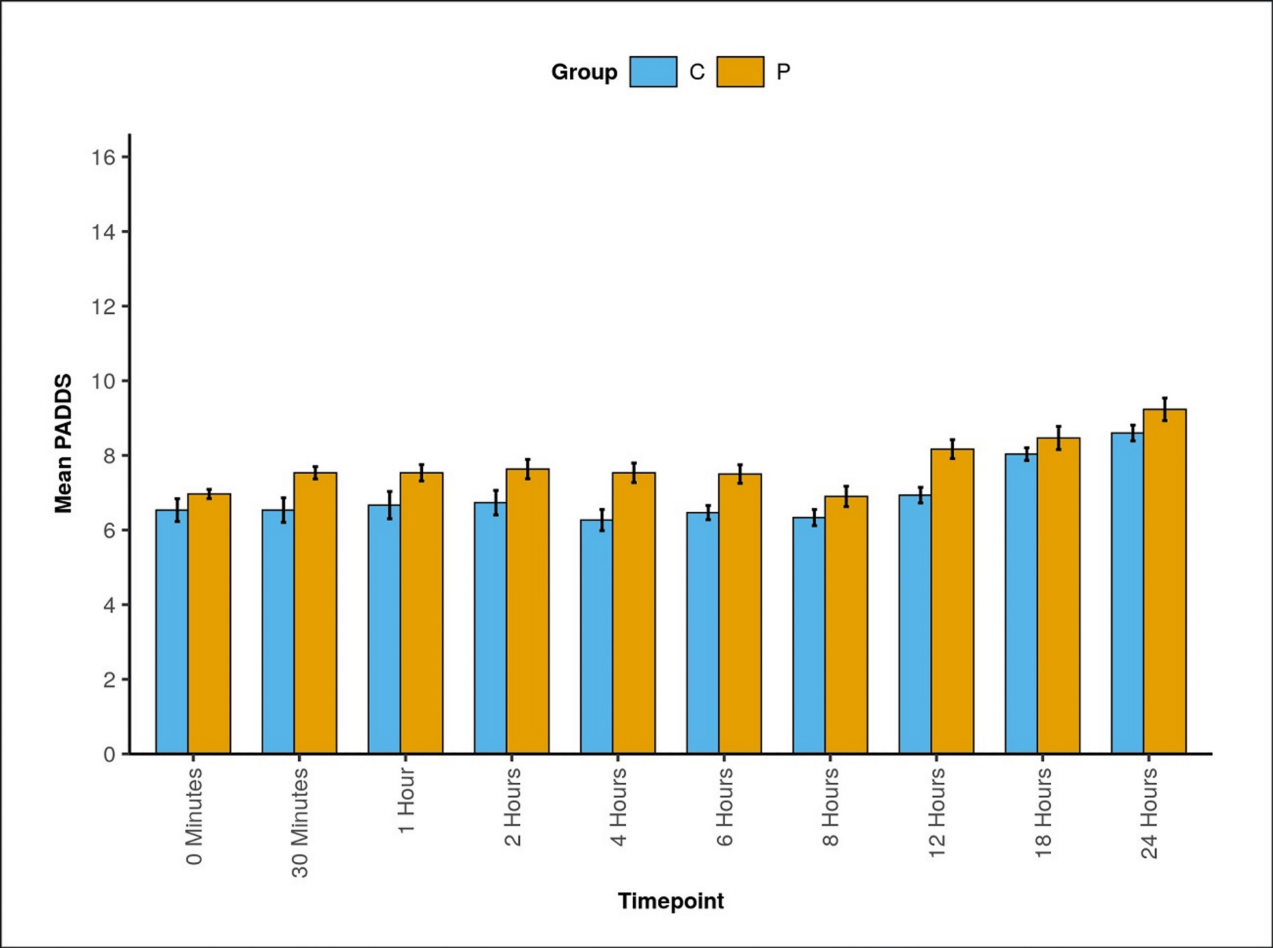
Bar diagram showing the change in PADSS (along with SD) over time between the two groups PADSS: Post-Anesthetic Discharge Scoring System; SD: standard deviation; Group C: caudal block group; Group P: pudendal block group

Significant differences in PADSS were observed between the two groups at all time points: 30 minutes, two hours, four hours, six hours, 12 hours, 18 hours, and 24 hours (Table 4, Figure 3).

**Table 4:** Comparison of the two groups in terms of change in PADSS over time PADSS: Post-Anesthetic Discharge Scoring System; SD: standard deviation; Group C: caudal block group; Group P: pudendal block group

Time point	PADSS		
	Group C: mean (SD)	Group P: mean (SD)	P-value (Wilcoxon-Mann-Whitney U test )
0 minutes	6.53 (1.68)	6.97 (0.67)	0.832 (U=463.000)
30 minutes	6.53 (1.80)	7.53 (0.90)	0.012 (U=297.000)
1 hour	6.67 (2.01)	7.53 (1.20)	0.069 (U=350.000)
2 hours	6.73 (1.80)	7.63 (1.43)	0.004 (U=286.000)
4 hours	6.27 (1.55)	7.53 (1.43)	<0.001 (U=216.000)
6 hours	6.47 (1.04)	7.50 (1.36)	<0.001 (U=210.000)
8 hours	6.33 (1.18)	6.90 (1.49)	0.090 (U=338.000)
12 hours	6.93 (1.14)	8.17 (1.39)	<0.001 (U=191.000)
18 hours	8.03 (0.93)	8.47 (1.70)	0.033 (U=309.500)
24 hours	8.60 (1.16)	9.23 (1.65)	<0.001 (U=221.500)
P-valuefor change in PADSS score over time within each group (Friedman test)	<0.001 (χ^2^=108.8)	<0.001 (χ^2^=121.3)	

Hemodynamic parameters were also assessed in both groups at various time points. Mean heart rate values were almost comparable between the two groups at all time points (p>0.05; Wilcoxon-Mann-Whitney U test) (Table 5). 

**Table 5:** Comparison of the two groups in terms of change in heart rate over time SD: standard deviation; Group C: caudal block group; Group P: pudendal block group; BPM: beats per minute

Time point	Heart rate (BPM)		
	Group C: mean (SD)	Group P: mean (SD)	P-value (Wilcoxon-Mann-Whitney U test)
Baseline	109.60 (13.44)	111.30 (12.90)	0.679 (U=421.5)
5 minutes	102.80 (10.75)	103.53 (8.34)	0.543 (U=408.5)
10 minutes	102.83 (14.14)	104.00 (12.75)	0.594 (U=413.5)
15 minutes	118.27 (21.00)	110.37 (10.41)	0.105 (U=560)
20 minutes	102.07 (12.00)	102.03 (10.96)	0.923 (U=443)
30 minutes	101.47 (12.29)	101.50 (10.65)	0.894 (U=440.5)
40 minutes	99.97 (9.89)	102.30 (10.37)	0.370 (U=389)
50 minutes	100.90 (8.45)	103.10 (9.05)	0.276 (U=376)
60 minutes	100.57 (8.61)	103.30 (10.29)	0.386 (U=391)
P-value for change in heart rate (BPM) over time within each group (Friedman test)	<0.001 (χ^2^=51.2)	<0.001 (χ^2^=42.7)	

Similarly, the mean systolic blood pressure (Table 6) and diastolic blood pressure (Table 7) of patients in both groups showed no significant difference (p>0.05; Wilcoxon-Mann-Whitney U test) at all measured time points. 

**Table 6:** Comparison of the two groups in terms of change in systolic BP over time SD: standard deviation; Group C: caudal block group; Group P: pudendal block group; BP: blood pressure

Time point	Systolic BP (mmHg)		
	Group C: mean (SD)	Group P: mean (SD)	P-value (Wilcoxon-Mann-Whitney U test)
Baseline	116.20 (10.67)	109.47 (10.21)	0.012 (U=620.5)
5 minutes	98.70 (15.17)	105.43 (13.57)	0.086 (U=333.5)
10 minutes	101.10 (12.89)	103.73 (10.67)	0.340 (U=385)
15 minutes	101.17 (14.02)	103.67 (10.68)	0.455 (U=399)
20 minutes	113.27 (10.29)	109.20 (7.63)	0.036 (U=592)
30 minutes	112.33 (9.99)	111.07 (9.19)	0.619 (U=484)
40 minutes	112.80 (10.08)	110.63 (9.35)	0.382 (U=509.5)
50 minutes	114.37 (8.87)	113.77 (7.93)	0.847 (U=463.5)
60 minutes	115.33 (10.01)	113.70 (8.72)	0.548 (U=491)
P-value for change in systolic BP (mmHg) over time within each group (Friedman test)	<0.001 (χ^2^=76.2)	<0.001 (χ^2^=51.1)	

**Table 7:** Comparison of the two groups in terms of change in diastolic BP over time SD: standard deviation; Group C: caudal block group; Group P: pudendal block group; BP: blood pressure

Time point	Diastolic BP (mmHg)		
	Group C: mean (SD)	Group P: mean (SD)	P-value (Wilcoxon-Mann-Whitney U test)
Baseline	72.43 (11.94)	71.37 (6.55)	0.250 (U=528)
5 minutes	58.80 (15.21)	63.00 (12.03)	0.225 (U=367.5)
10 minutes	58.90 (11.81)	60.27 (11.36)	0.534 (U=407.5)
15 minutes	57.60 (12.51)	59.37 (14.77)	0.437 (U=397)
20 minutes	71.63 (11.69)	70.03 (7.20)	0.202 (U=536.5)
30 minutes	74.10 (10.60)	71.10 (7.21)	0.103 (U=560.5)
40 minutes	72.83 (10.75)	69.23 (7.61)	0.059 (U=578)
50 minutes	72.87 (11.22)	68.37 (8.19)	0.059 (U=578)
60 minutes	74.33 (9.88)	69.27 (8.31)	0.039 (U=590)
P-value for change in diastolic BP (mmHg) over time within each group (Friedman test)	<0.001 (χ^2^=68.6)	<0.001 (χ^2^=47.0)	

## Discussion

The success rate for the first puncture was similar between the pudendal (73.4%) and the caudal (93.4%) groups. The overall block success rate was also similar between the pudendal (83.3%) and the caudal (90%) groups. A significantly lower amount of intraoperative fentanyl consumption, lower postoperative analgesia consumption, and longer first rescue analgesia were found among the pudendal block group than the caudal block group. Significant differences in the FLACC/VAS scores and PADSS scores were found between the groups over the period of time.

A recommended pain relief plan should offer safe and efficient pain management, minimize the body's stress response after surgery, and promote quicker recovery. A multimodal pain management approach is typically employed to attain these objectives [14].

The median first rescue analgesia time was significantly longer among the pudendal block group (17.5 hours vs 4.65 hours), which aligns with the findings of most previous studies. Ahmed et al., from their single-blinded trial from Egypt among children undergoing hypospadias surgery, reported the mean time for first rescue analgesia to be 18.5 hours in the pudendal block group and 10.56 hours in the caudal block group [11]. The first rescue time in the ultrasound-guided pudendal block is congruent with our study. In their study among 1-7-year-old Turkish children undergoing hypospadias procedure, Ozen and Ozen found that ultrasound-guided pudendal nerve block had a significantly longer effective block duration than the dorsal penile nerve block [15]. Earlier studies on applying pudendal block for children undergoing hypospadias surgeries also reported significantly better analgesia outcomes than the caudal block, even without ultrasound guidance [8,16]. Hence, in general, pudendal block yielded longer analgesic duration across the studies. This may be due to the fact that caudal space is very vascular in children and drug may be quickly absorbed into the circulation as compared to the ischiorectal fossa where pudendal block is performed. The variations observed in the present study as compared to the previous studies are likely due to differences in the type of drug, volume, and concentration of the local anesthetic and adjuvants used, operator and methodological variations, and a difference in the settings. In another study, Ravi et al. included children undergoing unilateral groin surgery where the average time to first rescue analgesia was more prolonged in ultrasound-guided ilioinguinal/iliohypogastric nerve block group (mean=253 minutes) as compared to that in the caudal group (mean=219.6 minutes) [13]. This shows that ultrasound-guided inter-fascial plane blocks have a longer duration of analgesia for lower abdomen surgeries in the pediatric population compared to conventional caudal blocks.

In the present study, intraoperative fentanyl consumption was significantly lower among the pudendal group than the caudal group, reiterating the better analgesic effect of pudendal block. Naja et al. reported the need for intraoperative fentanyl in the caudal block group as 10%, in comparison to 0% in the pudendal block group [8], echoing our findings. Okoro et al. reported a higher rescue morphine rate for ultrasound-guided pudendal block than the landmark-guided and conventional caudal block [12]. The difference in the findings might be because their study was a non-randomized, retrospective study of children under three years old. Thus, a parallel comparison is difficult to arrive at.

The success rate of the first puncture, an operational outcome, was statistically similar between the pudendal (73.4%) and caudal (93.4%) groups in the present study. Ultrasound guidance generally improves the success rate of the first puncture. Riaz et al. compared pediatric caudal block with ultrasound guidance versus the traditional landmark technique. They discovered that the ultrasound group had a greater first-try success rate (95%) than the landmark approach group (70.83%) [17]. Karaca et al. from Turkey reported a success rate at first puncture to be 92.5% in the ultrasound group and 66.2% in the conventional caudal group, which was highly statistically significant [9]. We experienced similar success with ultrasound-guided caudal block.

In contrast to intraoperative analgesia, the postoperative analgesia requirement of diclofenac was similar between the groups in the index study. Ahmed et al. reported a significantly lower acetaminophen requirement in the pudendal group than in the caudal group of patients [11]. Kendigelen et al. found that only three patients in the pudendal group needed additional analgesia within 24 hours after the surgery, while 100% of patients in the caudal group required analgesia within 24 hours, which was statistically significant [16]. The pain scores measured in the present study reported significantly lower pain in the pudendal block group than in caudal block patients till 20 hours following the procedure. Previous studies have also reverberated our findings, with better pain outcomes in the pudendal group [8,11,16].

The study's strength lies in the regional anesthesia technique's novelty and ultrasound-guided procedure usage. There is presently a paucity of robust literature supporting the novel ultrasound-guided transperineal approach. The present randomized controlled trial will add to the literature on using pudendal block as a safer alternative to analgesia for caudal block. Moreover, this study also adds valuable data to the limited studies on the pediatric population within an Indian context. However, the study has the following limitations: it is a single-center study with a limited sample size, and we have compared only two techniques using fixed doses. Therefore, it cannot be claimed that doses and drugs in the study provide the best analgesia efficacy, for the optimal analgesia doses used in the study may be higher and lower. The patients were not followed for more than 24 hours for analgesia outcomes and complications. Further follow-up could have resulted in a more relevant outcome for the study.

## Conclusions

Ultrasound-guided pudendal nerve block provides a longer duration and a better quality of analgesia in addition to a faster readiness for discharge as compared to caudal block in the pediatric population undergoing perineal surgeries under general anesthesia. Further, theoretically, the caudal block has potentially severe complications. Therefore, considering the findings of the present study, we recommend a wider adoption of the transperineal pudendal nerve block technique for perineal procedures in children. Additionally, further large-scale multi-centric studies with a robust study design, conducted among diverse patient populations in various clinical settings with longer follow-up duration, are warranted to improve the external validity of our findings.
